# Guardians of silence: transcriptional networks in T cell quiescence

**DOI:** 10.1038/s12276-025-01516-y

**Published:** 2025-08-04

**Authors:** Jin Ouk Choi, Yikhyeon Seo, Soo Seok Hwang

**Affiliations:** 1 School of Biological Sciences, Seoul National University, Seoul, Korea; 2 Institute of Molecular Biology and Genetics, Seoul National University, Seoul, Korea

**Keywords:** T cells, Gene regulation in immune cells

## Abstract

The maintenance of quiescence in T cells plays a pivotal role in averting undesired immune reactions and fostering immune homeostasis. Upon receiving external signals of cognate antigen and costimulatory molecules, T cells escape a quiescent state and rapidly proliferate within an exceedingly short timeframe. Nevertheless, for the majority of their lifespan, T cells remain inactive before stimulation, yet they are highly poised to future activation, implicating the presence of dynamic and intricate regulatory processes in a seemingly dormant state. While numerous extrinsic cues have been identified to induce T cell activation from a quiescence currently, intrinsic mechanisms governing T cell quiescence have received limited attention. Here we provide a comprehensive overview of multiple factors involved in T cell quiescence and their molecular mechanisms mainly in the context of transcriptional and post-transcriptional regulation. Given the intricate interplay between the control of T cell quiescence and a variety of diseases including autoimmunity, exhaustion and even tumor control, a thorough understanding of current insights into T cell quiescence affords us a valuable opportunity to advance our comprehension of T cell biology.

## T cell quiescence mechanisms unraveled for immune insights

The cells in adult mammals often enter a state called quiescence, where they stop dividing but can start again if needed. This study explores how T cells maintain quiescence and why it is important. Researchers found that T cells use various internal and external signals to stay in this nondividing state. They looked at how T cells use certain proteins and pathways to control their activity and readiness to respond to threats. The study involved reviewing existing research on T cell biology, focusing on the role of transcription factors like KLF2, FOXO1, FOXP1, BACH2 and BTG1/2. These factors help T cells remain inactive until needed by regulating genes involved in cell movement, survival, division and transcription. The findings show that maintaining quiescence is crucial for immune balance. Disruptions can lead to diseases like autoimmunity or cancer.

This summary was initially drafted using artificial intelligence, then revised and fact-checked by the author.

## Introduction

The majority of cells in adult mammalian are quiescent, defined as a state of reversible growth arrest. In the quiescent state, cells exit from normal cell cycle and remain in a nonproliferative state in G0 phase^1^. In addition to being devoid of cell division, smaller cell size, low metabolic activity and global suppression of RNA and protein synthesis are common hallmarks characterized in cellular quiescence^2^. However, quiescent cells are capable of resuming cell cycling upon certain stimuli from the extracellular environment while they retain their minimalistic characteristics^1,3^. Such reversibility confers a distinct feature from other types of nondividing cell, such as senescent or terminally differentiated cells, which are unable to proliferate permanently^3^. Unlike the terminological implication, it is now clear that quiescent cells are not inert but rather actively maintain their quiescent state by consistently expressing regulatory factors, which not only prevent proliferation and terminal differentiation but also prepare the cells for reentry into cell cycle^1,3^. These regulatory events commonly involve the inhibition of key regulators in cell cycle initiation, including cyclins and cyclin-dependent kinases (CDKs) or, alternatively, the induction of CDK inhibitors or retinoblastoma protein (Rb), which inhibits E2F, a transcriptional activator for cell cycle and division^1,3^. Of note, the concept of cellular quiescence has also been described in T lymphocytes (T cells), essential players in adaptive immune system. Naive and memory T cells in a resting state also actively maintain cellular quiescence but timely exit from a quiescent state and undergo proliferation and differentiation to optimize their functional effectiveness upon proper activating cues, such as cognate antigen engagement with T cell receptor (TCR)^4,5^. By contrast, aberrant regulation of cellular quiescence in T cells is closely related to pathological outcomes. Failure of quiescence exit leads to poor responsiveness to pathogens, as seen in immunodeficiency, whereas uncontrolled escape from a quiescence state results in autoimmune disorders by excessive activation of T lymphocytes^4^. Therefore, given the critical role of T cell quiescence in immune homeostasis, it will be worthwhile to understand T cell biology in the context of cellular quiescence. The maintenance of quiescence in T cells is sophisticatedly regulated by a variety of factors including metabolic changes, extrinsic factors and intrinsic regulators^4,6^. Although several features of T cell quiescence and its metabolic regulation have been previously reviewed^4^, the intrinsic regulation of quiescence has been poorly described. In this Review Article, we summarize the molecular mechanisms of intrinsic regulators specifically focusing on transcription factors and proteins regulating post-transcriptional regulations involved in maintaining T cell quiescence and highlight recent findings that elucidate the role of intrinsic regulation of this process.

## Overview of T cell quiescence

T lymphocytes originated from lymphoid progenitors, which are hematopoietic stem cells committed to lymphoid lineage^7^. Immature T lymphocytes, referred to as thymocytes, undergo developmental stages in the thymus, progressing from CD4^−^CD8^−^ double-negative to CD4^+^CD8^+^ double-positive and, finally, to CD4^+^ or CD8^+^ single-positive thymocytes^8^. T cell quiescence starts to build up in the transition from CD4^+^CD8^+^ double positive to CD4^+^ or CD8^+^ single positive and is fully established when T cells exit from the thymus as naive T cells^4,9^. Mature naive T cells circulate in the periphery via blood and lymphatic vessels, homing to secondary lymphoid organs (SLOs) where they receive essential survival signals and remain their quiescent state^10^. These quiescent naive T cells are metabolically and physiologically suppressed yet poised for activation upon recognition of a cognate antigen along with costimulatory signals and cytokines^4,11^.

## Extrinsic regulation of T cell quiescence

**Fig. 1:**
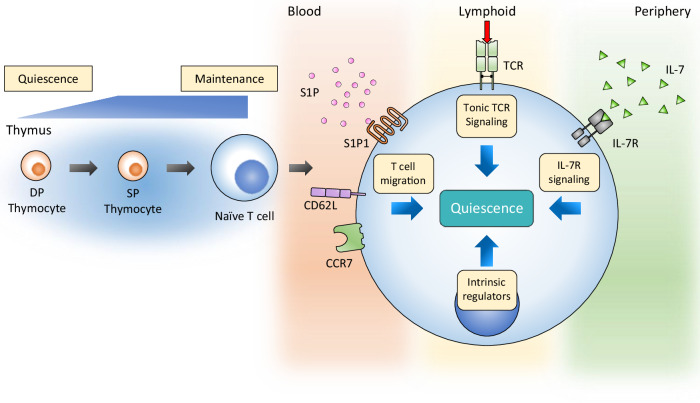
Quiescence begins to be established in the DP thymocyte stage and completed in fully matured naive T cells. During circulation, multiple factors contribute to the maintenance of quiescence in naive T cells. Trafficking molecules (S1P1, CD62L and CCR7) guide T cell circulation, leading T cells to encounter diverse factors involved in the maintenance of quiescence. S1P, abundant in blood, directs egress of T cells from lymphoid organs and promote the survival of quiescent T cells via its receptor S1P1. Tonic TCR stimulation occurs in lymphoid organs by the engagement of self-peptide bearing MHC molecules and facilitate T cell quiescence. High level of IL-7 in periphery also supports the survival of T cells and prevent quiescence exit through IL-7R. Intrinsic regulators programmed in quiescent T cells coordinate these extrinsic signals for optimal maintenance of quiescence. DP, CD4^+^CD8^+^ double positive; SP, CD4^+^ or CD8^+^ single positive.

Under normal conditions lacking activating stimuli, T cell quiescence is maintained by intrinsic programs along with extrinsic cues, including the cytokine interleukin 7 (IL-7) (ref. ^12^), tonic TCR signaling^13^ and other regulatory signals from the lymphoid niche, fostering long-term survival and proper positioning of naive T cells (Fig. 1). The maintenance of peripheral naive T cell pool in a quiescent state relies on signals from IL-7, which is essential for T cell survival and homeostatic proliferation^14^. IL-7, constitutively provided by stromal cells in peripheral lymphoid organs, primarily influences T cells through their expression of the IL-7 receptor (IL-7R), particularly the IL-7 α-chain (IL-7Rα; CD127)^15^. Numerous studies have reported that the survival of mature and memory T cells is profoundly compromised by IL-7 deficiency or blockade^16,17^. Signaling through JAK3-STAT5 pathway downstream of IL-7R induces the antiapoptotic molecule Bcl-2 (ref. ^14^) and enhances the protein stability of CDK inhibitor p27^Kip1^, preventing cell cycle entry^18^. This function of IL-7 is closely linked to the microenvironment of lymph nodes and the spleen, where quiescent T cells express CD62L (L-selectin) and C–C chemokine receptor 7 (CCR7), facilitating their migration and localization within peripheral lymphoid organs. CD62L, also known as L-selectin, promotes T cell adhesion to high endothelial venules and aids in homing to lymph nodes. CCR7, a chemokine receptor for CCL19 and CCL21, guides T cells from the cortex to the medulla of the thymus and to the T cell zones in lymph nodes during migration. Upon entering the lymph node microenvironment, fibroblastic reticular cells provide essential survival signals, including IL-7, to maintain T cell quiescence and homeostasis^19^. Moreover, sphingosine-1-phosphate receptor 1 (S1P1) serves a critical function in T cell egress by binding to sphingosine-1-phosphate (S1P), which is abundant in the outer region of the thymus; its deficiency results in T cell lymphopenia in peripheral lymphoid organs^20^. S1P promotes naive T cell survival by supporting energy provision for T cell migration through mitochondrial regulation^21^. Tonic TCR stimulation occurs when TCR engages with self-peptide-bearing major histocompatibility complex (MHC) molecules presented by antigen-presenting cells in SLOs^13,22^. Unlike strong TCR signaling induced by cognate antigens, which leads to quiescence exit and T cell activation, tonic TCR stimulation activates several intrinsic regulators, such as FOXO1, that sustains T cell quiescence by promoting IL-7R expression, essential for IL-7 signaling^13^.

## Metabolic regulation of T cell quiescence

Naive T cells remain in a quiescent, nonproliferative state until they encounter proliferative TCR stimulation. During this period, they maintain long-term survival while continuously circulating between SLOs and the bloodstream in preparation for potential antigenic challenges. To adapt to conditions of limited energy availability, naive T cells exhibit low metabolic activity^23^. Rather than relying on glycolysis, they primarily utilize oxidative phosphorylation through pyruvate and fatty acid oxidation that supports their longevity under energy-restricted conditions^24^. TCR stimulation and CD28 costimulatory signals induce a dramatic metabolic reprogramming, followed by enhanced anabolic processes including the synthesis of mRNA, lipids and proteins to support cell growth and extensive proliferation^25^. To rapidly meet the increased energetic and biosynthetic demands, activated T cells shift their metabolism from oxidative phosphorylation to glycolysis and glutaminolysis. Specifically, glycolysis facilitates the rapid generation of ATP through the conversion of glucose to pyruvate. Increased expression of lactate dehydrogenase A (LDHA) promotes the conversion of pyruvate to lactate, regenerating NAD⁺ from NADH and thereby sustaining high glycolytic flux. Concurrently, glutamine is metabolized through glutaminolysis, yielding glutamate and α-ketoglutarate, which results in anaplerotic reaction replenishing intermediates of TCA cycle, which supports the biosynthesis of amino acids, lipids and nucleotides, including purines and pyrimidines^26^.

Following T cell activation, the expression of nutrient transporter proteins such as GLUT1, SLC1A5 and SLC7A5 is markedly upregulated, facilitating increased uptake of extracellular glucose and glutamine^4^. Concurrently, the expression of key metabolic enzymes involved in glycolysis and glutaminolysis is also enhanced. These metabolic adaptations are largely driven by activation of the PI3K–Akt–mTOR pathway, which acts in concert with Src family kinases, promoting the activity of CDKs, leading to cell proliferation through reentry into cell cycle^27^. mTORC1 plays a diverse role in the exit from quiescence by regulating ribosomal biogenesis through activation of p70S6K and suppression of translational repressors such as 4E-BP, thereby promoting overall protein synthesis^28^. Furthermore, mTORC1 induces c-Myc expression, a positive regulator of interphase CDKs and cyclin expression^29^. Myc drives the metabolic shift in T cells from FAO and oxidative phosphorylation to glycolysis and glutaminolysis, promoting effector T cell growth and activation. While mTORC1 enhances Myc synthesis via eIF4E, in turn, Myc upregulates amino acid transporters to boost glutamine uptake, reinforcing mTORC1 signaling in a feedback loop that accelerates T cell prolife ration and activation^23^. Furthermore, mTORC1 promotes localization of SREBP1/2 into the nucleus, which transcriptionally activates proteins mediating cholesterol and fatty acid biosynthesis^30^. These axes collectively lead to cell growth and enhancement of cellular metabolism, supporting the robust growth and proliferation of activated T cells.

In naive T cells, tight regulation of the PI3K–Akt–mTORC1 checkpoint is essential to prevent excessive metabolic activation, spontaneous T cell activation and the potential development of autoimmunity. Key negative regulators of this pathway include PTEN and TSC proteins. PTEN antagonizes PI3K activity by converting phosphatidylinositol^3–5^-trisphosphate (PIP3) to phosphatidylinositol^4,5^-bisphosphate (PIP2), serving as a negative regulator of AKT signaling^31^. In CD4⁺ T cells, PTEN deficiency does not necessarily result in spontaneous activation; however, it lowers the TCR signaling threshold, allowing robust AKT activation even in the absence of CD28 costimulation^32^. TSC2 functions as a GTPase-activating protein (GAP) for the small GTPase RHEB, which directly activates mTORC1 (ref. ^33^). In the absence of TSC2, naive T cells exhibit constitutive activation of mTORC1, as evidenced by increased phosphorylation of downstream targets S6K and 4E-BP1. These cells tend to be hyperactivated, highly glycolytic phenotype even without full activation and fail to efficiently transition into memory T cells^34^. In the absence of TSC1, which stabilizes TSC2, T cells become hypersensitive to TCR stimulation, leading to rapid and aberrant activation, premature cell cycle entry, elevated reactive oxygen species production and increased expression of proapoptotic factors such as BIM, ultimately resulting in increased cell death^35^.

Thus, T cells maintain their quiescent state via negative regulator of their critical metabolic checkpoints, which is critical for appropriate and timely activation. In this context, transcription factor FOXO1 is target of AKT-mediated phosphorylation and thereby degradation, which regulating the aforementioned expression of genes extrinsic factors for T cell quiescence including IL-7Rα, CCR7 and CD62L^36^. Therefore, both extrinsic signals and intrinsic regulators are intricately interconnected to maintain the quiescence and long-term survival of naive T cells.

## Intrinsic regulation of T cell quiescence mediated by transcription factors

Although diverse extrinsic stimuli and metabolic regulators have been known to control quiescence in T cells, these are intertwined with many intrinsic factors to optimally maintain T cell quiescence. Both extrinsic cues inducing quiescence or the absence of activating stimuli enable T cells to continuously express specific gene programs associated with intrinsic regulators. Currently, several intrinsic factors have been reported that are specialized for maintaining T cell quiescence. Most of these factors are multifunctional, and their control extends beyond the maintenance of naive T cells. Among these intrinsic regulators, transcription factors play a central role in mastering T cell quiescence downstream of extrinsic and intrinsic signaling. Hereby, we introduce the transcription factors KLF2, FOXO1, FOXP1 and BACH2 as pivotal in maintaining T cell quiescence.

## KLF2, a regulator of T cell trafficking for quiescence maintenance

**Fig. 2:**
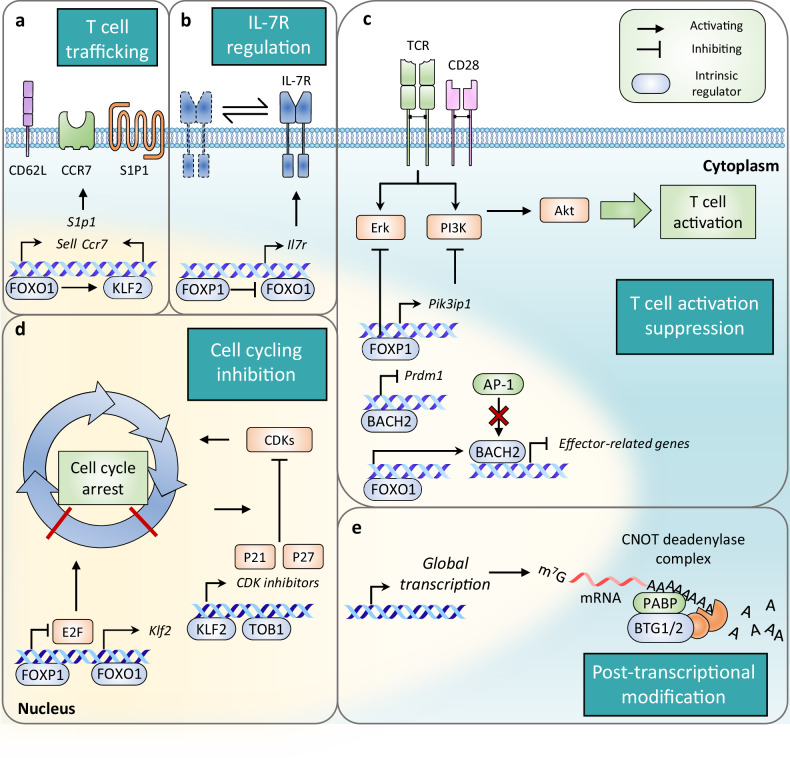
Molecular interactions of intrinsic regulators in the maintenance of T cell quiescence. The summarized molecular mechanisms of intrinsic regulators in T cell quiescence. The regulation of T cell trafficking molecules (**a**) and IL-7R (**b**), suppression of T cell activation (**c**), cell cycling inhibition (**d**) and downregulation of global mRNA abundance (**e**). In **a**, FOXO1 and KLF2 induce the transcription of T cell trafficking molecules (CD62L, S1P1 and CCR7), which are essential for proper migratory function of naive and memory T cells to maintain a quiescent state. In **b**, FOXO1 promotes IL-7 signaling by inducing IL-7R expression. FOXP1 inhibits IL-7R expression by antagonizing FOXO1, ultimately prevents excessive IL-7R signaling. Thus, the duration of IL-7R signaling is balanced by both FOXO1 and FOXP1. In **c**, TCR-mediated activating signals are suppressed by FOXP1 activity. FOXP1 directly inhibits Erk and induce the transcription of *Pik3ip1*, an inhibitor of PI3K. The expression of genes targeted by AP-1, an important transcription factor for effector function of activated T cells, is competitively blocked by BACH2 which share same binding sites of AP-1. Also, BACH2, induced by FOXO1 inhibits *Prdm1* expression. In **d**, FOXP1 blocks the expression of E2F, a key molecule for cell cycle progression. FOXO1 upregulates *Klf2* expression, and KLF2 and TOB1 also induce CDK inhibitors, P21 and P27, which prevent cell cycling by inhibiting CDK activity. In **e**, the global abundance of mRNA is downregulated by BTG1/2 expressed in a quiescent T cell. BTG1/2 bind to PABP on poly(A) tail of mRNA and recruit CNOT deadenylase complex. The interaction between BTG1/2 and CNOT complex enhance deadenylase activity, resulting in mRNA degradation by shortening poly(A) tail. PI3K, phosphoinositide-3-kinase; IL-2, interleukin 2.

Krüppel-like factor 2 (KLF2), also known as lung Krüppel-like factor (LKLF), is a zinc-finger transcription factor of KLF family which is the first transcription factor identified as being involved in the quiescence of T lymphocytes^37^. Its expression is generally observed in mature thymocytes including naive and memory T cells but diminishes upon TCR stimulation^38^. KLF2 is expressed during the maturation of single-positive T cells in the thymus, and KLF2-deficient T cells were found to be highly apoptotic^38^. Given that the absence of KLF2 is embryonically lethal, early studies of KLF2 utilized a chimeric RAG2^−/−^KLF2^−/−^ mouse model, in which KLF2^−/−^ embryonic stem cells were injected into *Rag2*^−/−^ blastocysts. This allowed the chimera to possess KLF2^−/−^ lymphocytes without developmental defects^38^. A reduced number of peripheral T cells was observed in chimeric RAG-2^−/−^KLF2^−/−^ mice, and KLF2-deficient T cells displayed activated phenotype along with nonproliferative features^38^. Subsequently, several studies involving overexpression of KLF2 in Jurkat T cells demonstrated that KLF2-induced Jurkat T cells exhibited a quiescent phenotype. In addition, these studies suggested a functional pathway through which KLF2 induces p21^WAF1/CIP1^, a CDK inhibitor, and negatively regulates Myc, a multifunctional transcription factor involved in cell growth and division^37,39,40^ (Fig. 2d). Mechanistically, Myc promotes CDK2 activity by inducing transcription of *cdc25a* and repressing *Gadd45* (refs. ^41,42^), a growth inhibitory gene. Therefore, its suppression by KLF2 likely contributes to maintaining a quiescent state. In addition, overexpression in tumor cell lines, such as NSCLC^43^ and breast cancer^44^, suppresses their proliferation and mediates cell cycle arrest at the G0/G1 phase, thereby underscoring its role as a tumor suppressor.

However, the effects of KLF2 on cell cycle regulation, observed through overexpression in activated T cells or Jurkat cell line, have been challenged by more physiologically relevant and sophisticated experiments. One of these experiments demonstrates that overexpression at physiological levels results in the deceleration of the proliferative expansion, rather than quiescence mediated by regulation of Myc and p21^WAF1/CIP1^(ref. ^45^), while defining the primary role of KLF2 as regulating molecules involved in the trafficking of naive T cells^45–48^. As thymocytes complete their maturation in the thymus, single positive CD4^+^ or CD8^+^ T cells egress from the thymus and home to SLOs with assists of several T cell trafficking molecules^45,46,49^. Carlson and colleagues first reported impaired expression of T cell trafficking molecules—S1P1, CD62L, CCR7 and β_7_ integrin—in RAG-2^−/−^KLF2^−/−^ mice^50^. This view is further supported by studies using overexpression and Cre-mediated knockout systems^47,51,52^, which demonstrate that KLF2 directly induces the transcription of CD62L and S1P1 (Fig. 2a). Subsequently, another study has shown that KLF2 is required to suppress the expression of various chemokine receptors associated with T cell migration during inflammatory responses, including CXCR3 (refs. ^45–47^), where abrogation of migratory receptors leads to the abnormal distribution of T cells across SLOs and peripheral tissues^47^. Moreover, KLF2-deficient T cells showed increased levels of cytokine production (IL-4, IFN-γ and TNF) whether through direct or indirect regulation by KLF2 (ref. ^52^). Taken together, the findings show that KLF2-deficient T cells promote an autoimmune phenotype such as increased serum IgE level and lymphopenia^47,50,52^. The broad role of KLF2 in immune responses is mediated through its direct regulation of molecules related to T cell chemotaxis, such as CD62L and S1P1. Nevertheless, the role of KLF2 in contexts beyond its control of T cell trafficking and needs to be further studied.

## FOXO1, an integrator of transcriptional networks in T cells

FOXO proteins, which belong to the forkhead box (FOX) family, are comprised of four subfamily members in mammals: FOXO1, FOXO3, FOXO4 and FOXO6 (ref. ^53^). FOXO proteins can directly regulates target gene expression using winged helix forkhead DNA-binding sites that interacts with the consensus motif 5′-TTGTTTAC-3′ (ref. ^53^). In addition, FOXO proteins cooperate with other binding partners—C/EBPβ, STATs, Smads, p300 and various nuclear receptors^54^—implying a diverse range of roles for FOXO-mediated regulation. The activity of FOXO is controlled by phosphorylation, which determines its cellular localization and degradation^53^. For instance, PI3K–Akt signaling can phosphorylate FOXO proteins, hindering their ability to bind DNA and inducing their translocation from nucleus to cytoplasm^53^. Among FOXO subfamily members, the expression of FOXO1 is specifically induced during T cell maturation^55^ and is known to contribute T cell quiescence while FOXO3 and FOXO4 are also expressed in T cells. Similar to the function of KLF2, FOXO1 can regulate T cell trafficking molecules, as first reported in a study where FOXO1 overexpression in Jurkat T cells resulted in the high expression of CD62L, S1P1 and CCR7, whereas T cells from FOXO1-deficient mice showed impaired expression of these molecules^56^. Intriguingly, FOXO1 can directly binds to the *Klf2* promoter to regulate its expression^56^, which regulates genes involved in T cell trafficking as discussed in the previous section (Fig. 2d). The fact that FOXO1 could upregulate their expression even when KLF2 is partially knockdown^56^, and its ability to directly bind to the genomic loci of *Sell* and *Ccr7*(ref. ^57^)(Fig. 2a) indicates that FOXO1 may regulate their expression directly, although their expression is primarily regulated by KLF2-dependent manner^45^.

However, despite the lack of details, it seems apparent that FOXO1 may facilitate proper migratory function of T cells in a KLF2-independent manner in other immune contexts, where FOXO1 regulates *Sell* and *Ccr7* expression without substantial changes in *Klf2* expression^58^. Notably, various studies have demonstrated that the expression of IL-7Rα in steady state T cells is directly regulated by FOXO1, as evidenced by the use of a conditional knockout system targeting the *Foxo1* gene in T cells^36,59^. As shown in these studies, FOXO1 deficiency in T cells results in the decreased expression of IL-7Rα at both the mRNA and protein levels^36,59^. Regulation of IL-7Rα by FOXO1 was associated with direct binding to the evolutionary conserved noncoding region located 3.7 kb upstream of *Il7r* gene translation start site, which indicates that IL-7R is one of the targets of FOXO1 (refs. ^36,59^). In addition to regulating IL-7Rα, the absence of FOXO1 was associated with an increased number of activated populations of CD44^+^CD62L^−^ in CD4^+^ and CD8^+^ T cells, as well as more differentiated T cells expressing cytokines such as IFN-γ, IL-4, IL-17 or IL-10 (ref. ^59^). Consistently, chimeric mice reconstituted with FOXO1-deficient bone marrow spontaneously developed severe colitis, accompanied by a higher proportion of effector T cell populations^59^, further supporting the role of FOXO1 in control of T cell tolerance and homeostasis. Furthermore, the integrative role of FOXO1 has been proposed in a study utilizing a FOXO1-AAA mutant, which is continuously expressed in T cells to enable FOXO1 to maintain its activity, which normally diminishes after T cell activation^60^. Prolonged expression of FOXO1 resulted in imbalanced cell growth and proliferation, which was associated with the suppression of newly defined targets by FOXO1, including Myc, ribosome biogenesis and cholesterol biosynthesis^60^. Moreover, FOXO1 decreased mTORC activity, indicating the important role of FOXO1 in the cell intrinsic coordination of growth and proliferation. A recent study of naive and memory CD8^+^ T cells has also suggested that FOXO1 inhibits AP-1 family transcription factor, which is responsible for the upregulation of genes involved in effector functions such as *Ifng* (Fig. 2c). This inhibition occurs directly by FOXO1 binding to the genomic loci of AP-1 transcription factor family, or indirectly by inducing BACH2 in naive and memory CD8^+^ T cells^57^. BACH2, which will be further elaborated upon in a subsequent paragraph, additionally suppresses the activity of AP-1 transcription factors by occupying their enhancer regions. Furthermore, the study has revealed a correlation between FOXO1 and cellular senescence, which resembles cellular quiescence but represents a permanent arrest in the G0 phase^57^. In the absence of FOXO1, memory T cells not only displayed excessive activation but also exhibited a senescent phenotype^57^. Of note, the expression of FOXO1 was diminished in old (18–24 months) mice compared with young controls, along with TCF1, an essential regulator in memory T cell maintenance, which suggests that FOXO1 also contributes to maintaining reversibility during a quiescent state by balancing between quiescence and senescence^57^.

## FOXP1, a balancer for IL-7 and TCR signaling in T cells

Forkhead box protein P1 (FOXP1), a member of large FOX transcription factor family, is expressed in various cell types and is an essential transcription factor for B lymphocytes development^61,62^. In the context of T lymphocytes, it has also been reported that the conditional loss of FOXP1 in CD4^+^CD8^+^ double-positive thymocytes resulted in single-positive thymocytes becoming more sensitive, accompanied by increased cytokine production and apoptosis^63^, indicating that FOXP1 plays a critical role in the development of quiescent naive T cells. In a study utilizing inducible deletion of *Foxp1*, mature naive T cells deficient in FOXP1 exhibited effector phenotypes and demonstrated increased proliferation, which was dependent on IL-7 (ref. ^64^). The study has demonstrated that FOXP1 negatively regulates the expression of IL-7R by antagonizing FOXO1 activity. FOXP1 binds to forkhead-binding site of *Il7r* gene enhancer region, which is also targeted by FOXO1, thus antagonizing FOXO1’s activity^64^. Although FOXO1 promotes T cell quiescence by enhancing the survival and homeostatic proliferation through the upregulation of IL-7R, thereby enhancing IL-7 signaling, the role of FOXP1 in T cell quiescence appears paradoxical given that it downregulates IL-7R. Prolonged signaling through IL-7R induces rather proliferation of naive T cells and enhances IFN-γ production, leading to cytokine-induced cell death, which was observed in a study with naive CD8^+^ T cells continuously expressing IL-7R^12^. For the maintenance of quiescent T cells, IL-7 signaling should be intermittent, and this should occur in conjunction with tonic TCR signaling^12^. Therefore, the proper balance in the regulation of IL-7R by FOXP1 and FOXO1 is critical for the homeostasis of quiescent T cell pool through the fine-tuning of IL-7 signaling (Fig. 2b). In addition to control of IL-7R, several studies have further defined the role of FOXP1 in T cell quiescence that regulates a variety of signaling pathways associated with T cell activation, differentiation, growth and proliferation.

The MEK/ERK pathway is one of the critical signaling pathways involved in TCR stimulus, leading to the T cell activation. In the absence of FOXP1, CD8^+^ T cells exhibited enhanced ERK phosphorylation upon TCR stimulation, highlighting the role of FOXP1 in limiting the activity of ERK and its downstream pathway^64^. Actually, MEK/ERK pathway inhibits the transcription of *Klf2* after TCR engagement^45^, which suggests another regulation network among transcription factors related to quiescence. FOXP1 also downregulates the PI3K–Akt–mTOR pathway by directly inducing the expression of PI3K interacting protein 1 (*Pik3ip1*)^65^, a negative regulator of upstream of PI3K, which in turn leads to decreased Akt phosphorylation, leading to the suppression of T cell activation^65,66^ (Fig. 2c). Moreover, FOXP1 negatively modulates cell cycle progression through the downregulation of E2F transcription factor and cell cycle gene expression^65^. Its inducible deletion in naive CD8^+^ T cells cultured with IL-7 leads to the increased mRNA levels of *E2f1*, *E2f3* and *Cdk1*, as well as markedly enhanced IL-7-driven homeostatic proliferation. This regulation of cell cycle occurs independently of the retinoblastoma protein (Rb)^65^, a well-known tumor suppressor that inhibits E2F function, suggesting that FOXP1 controls E2F at the transcriptional level to ensure T cell quiescence (Fig. 2d).

In summary, FOXP1 regulates quiescence by inhibiting key TCR signaling pathways and through genes associated with cell cycle progression. The role of FOXP1 in memory T cell and other immune contexts, as well as the relevance of its function in maintaining quiescence, remains to be further elucidated.

## BACH2, a gatekeeper of effector T cell differentiation by antagonizing AP-1

BACH2 acts as a transcriptional repressor, which belongs to the basic leucine zipper (bZIP) transcription factor family. It was previously known to function in B cell somatic hypermutation and antibody class switching^67^, and it was suspected to have functions in T cell homeostasis because its genetic mutations lead to various autoimmune phenotypes^68–70^, such as asthma^71^ and multiple sclerosis^72^. The expression of BACH2 remains high throughout T cell maturation and in naive T cells, but it is progressively downregulated as T cells become activated—from central memory T cells to the effector phase—with the lowest levels observed in terminally differentiated T cells^73^. Although T cell development appears normal in germline knockouts of BACH2, a decrease in T cell numbers has been observed in the spleen and lymph nodes. This reduction is attributed to the decreased ratio and numbers of naive T cell population, which contributes to systemic inflammation^74^. Moreover, BACH2 deficiency in mixed bone marrow chimera mice results in the impaired central memory and memory precursor effector cells, as well as an increased population of short-lived effector cells in a virus infection model, which indicates that BACH2-knockout disrupts naive T cell homeostasis, promotes terminal differentiation and leads to the increased apoptosis^73^. In BACH2-deficient mice, naive T cells exhibit heightened T cell differentiation and function, marked by increased production of cytokines such as IFN-γ, IL-4 and IL-17, along with their master transcription factors, despite the unchanged rates of proliferation during early T cell activation^69,73,74^. Furthermore, successive rounds of cell cycle following naive T cell activation lead to increased apoptosis in BACH2-deficient cells, accompanied by reduced antiapoptotic BCL-2 protein family, characterizing increased effector differentiation^73^. Disruption of quiescence in BACH2-deficient T cells stems from its control over genes related to effector lineages. For instance, the chemokine receptor CCR4, which has CCL21 as its ligand and is typically upregulated in activated T cells to facilitate recruitment to inflammatory sites, is highly expressed in naive T cells lacking BACH2 (ref. ^74^). Chromatin immunoprecipitation followed by sequencing (ChIP-seq) analysis of activated T cells found that BACH2 binds to the genes such as *Gata3*, *Irf4*, *Nfil3*, *Il12rb1 and Il12rb2*, which are related to the effector differentiation and lineage, thereby suppress their expression^69^. BACH2 also binds to the *Prdm1* locus to repress its expression, which encodes BLIMP-1, a major regulator of T cell effector differentiation (Fig. 2c). The ability of BACH2 to regulate effector differentiation is attributed to the attenuation of TCR-driven gene expression by AP-1 transcription factor family, which shares a common transcription factor binding motif with BACH2 (ref. ^73^). It allows BACH2 to occupy the same binding sites before TCR engagement, thereby limiting the access of AP-1 transcription factors and reducing their target gene expression. Within several hours of naive T cell activation, there is a increase in gene expression regulated by AP-1 transcription factor family, such as *Ifng* and *Gp49a*, as well as effector-related genes including *Cd44*, *Ccl1*, *Ccl19* and *Lta*. In the absence of BACH2, there is an increased occupancy of JunD, a member of AP-1 transcription factor family, at the TCR-driven gene enhancer loci, resulting in enhanced expression of its target genes, thereby exhibiting an activated phenotype.

Super-enhancers are large clusters of transcriptional enhancers densely packed with transcription factors and regulatory proteins that exhibit enhanced transcriptional activity. Characterized by high H3K27Ac signals and substantial p300 occupancy, they play a crucial role in defining cellular identity^75^. BACH2 binds to 26% of super-enhancer regions in CD4^+^ T cells, repressing crucial T cell identity genes, including cytokines and their receptors. Besides controlling genes in super-enhancers, *Bach2* genomic locus itself is one of the most prominent super-enhancer regions, suggesting the critical role of BACH2 in the regulation of T cell homeostasis and functions^76^. A recent study found that BACH2 also participates in maintaining the stemness of memory T cell population, where its deficiency was shown to impair TCF1^hi^Tim3^lo^ stem-like memory population^77^. In addition, gRNA-mediated knockout of BACH in this population led to the upregulation of PD-1 and Tim-3 as well as genes related to terminal exhaustion, such as *Gzmb and Klrg1*. On the contrary, overexpression of BACH2 suppresses the expression of effector-related molecules^74^ and reinforces the stemness property of memory population^77^. Overall, BACH2 helps maintain T cell quiescence by repressing the expression of TCR-driven genes and effector-signature genes.

## Post-transcriptional regulation in control of T cell quiescence

Target gene expression in T cells is actively regulated by post-transcriptional mechanisms such as RNA splicing, RNA degradation and RNA modification across various cellular stages including naive states, memory formation and differentiation processes^78^. However, the role of post-transcriptional regulation associated with T cell quiescence has recently been emphasized. Splicing factor SRSF1 has been found to regulate quiescence by inhibiting the hyperactivity of T cells^79^. SRSF1 upregulates the expression of PTEN, a critical negative regulator of the PI3K–Akt pathway, which in turn represses mTORC1 activity. Consequently, this leads to the downregulation of T cell activation. In the absence of SRSF1, T cells become hyperactivated due to enhanced mTORC1 signals. In patients with systemic lupus erythematosus, a low abundance of SRSF1 and an increase in proinflammatory cytokine have been linked observed and overexpression of SRSF1 could reverse this phenotype and rescue the hyperactivated T cell state.

*N*6-methyladenosine (m^6^A) mRNA methylation, one of the most abundant RNAmodifications, also plays a critical role in T cell homeostasis by modulating the IL-7/STAT5/SOCS signaling pathways in naive T cells^80^. In mice deficient in *Mettl3* (the m^6^A writer), elevated levels of suppressor of cytokine signaling (SOCS) proteins were observed. This increase was attributed to the absence of m^6^A-mediated degradation of *SOCS* mRNA, which normally facilitates its recognition and subsequent breakdown. Upregulated SOCS proteins disrupted IL-7-driven T cell differentiation and cytokine signaling, resulting in disrupted T cell expansion and homeostasis.

Recently, long noncoding RNA have also been found to modulate T cell quiescence. Long-interspersed element-1 (LINE1) has noncanonical splicing variants that are integrated into the genome by retro-transposition where its integration mediates target gene repression through PABP1. LINE1 is highly expressed in quiescent naive and memory T cells but can be expressed when T cells become exhausted, thereby limiting its effector function, a novel mechanism to restrain T cell activity^81^. Previously outlined were the recently discovered post-transcriptional regulators that control naive T cell quiescence and homeostasis. The subsequent discussion will be focused on BTG1 and BTG2, which have been recognized as antiproliferative proteins and recently identified as post-transcriptional regulators regulating T cell quiescence.

## BTG1 and BTG2, post-transcriptional regulators of mRNA stability in quiescent T cells

The recent study has proposed BTG1 and BTG2 (BTG1/2) as novel regulators of T cell quiescent^82^. BTG1/2 are specifically expressed in quiescent T lymphocytes, both naive and memory subsets, and their expression is diminished upon activation^82^. BTG1/2 contribute to the active maintenance of quiescence, as BTG1/2 deficient T cells have displayed higher expression of activation markers such as such as CD25, CD44 and CD69 and more proliferative^82^. BTG1/2 are members of the BTG/TOB family that shares a highly conserved N-terminal BTG domain, providing a module for protein–protein interaction^83^. Among the members in BTG/TOB family, BTG1/2 are highly similar to each other and have unique feature of an additional eight to ten amino acids in N-terminal of the BTG domain, distinct from other members^83^. Through this domain, BTG1/2 interact with diverse transcription factors, facilitating their DNA binding and stimulating transcription factor activity, as reported in myoblast cells^84^. Notably, BTG proteins are also known for their antiproliferative activity in various cell types by modulating mRNA turnover. Several studies have revealed that BTG1/2 interact with previously mentioned deadenylase complex of CCR4-NOT (CNOT) and poly(A) tail binding protein (PABP), promoting mRNA degradation by deadenylation of poly(A) tail^83,85,86^. In detail, BTG1/2 bind to RRM domain of cytoplasmic poly(A)-binding proteins 1 (PABPC1) through their BTG domain, which is sufficient to induce mRNA deadenylation^87^. Of note, BTG1/2 present in quiescent T cells, continuously downregulate the global abundance of cytoplasmic mRNA through interaction with the PABP-CNOT7 deadenylase complex^82^. Indeed, CD4^+^ T cells from BTG1/2-double-knockout mice exhibited the longer poly(A) tails in global mRNA^82^. Consistent with this, the overall level of mRNA was increased along with enhanced protein expression, rendering T cells to overly sensitive to activation signal. Thus, the process by which BTG1/2 maintain T cell quiescence appears to provide quiescent T cells with advantages by harnessing premade mRNA for quick response, instead of complete shut-down of mRNA synthesis corresponding to a quiescent state^82^ (Fig. 2e). A recent study found out that in patients with B cell lymphoma, BTG1Q36H, the missense mutation of BTG1, was associated with a poor clinical outcome^88^. BTG1Q36H mutant B cells are more activated and exhibit increased proliferative burst following the germinal center reaction. Mutant B cells undergo an enhanced Myc-dependent program and upregulate mTORC1, which are important in controlling the biosynthetic preparedness of growth program. More specifically, BTG1 may targets mRNA of Myc target genes and Myc itself, suppressing its post-transcriptional regulation, which is lost and leads to enhanced polysome loading in the mutant B cell. Although further studies are needed to elucidate the same mechanism is applicable in T cells, existing research has demonstrated that BTG1/2, predominantly found in lymphoid organs and leukocytes^82^, may have distinct roles in the immune system compared with other members of the BTG/TOB family. Taken together, these findings offer new mechanistic insights into how T cells maintain reversibility during their quiescence by controlling post-transcriptional modifications. A recent study in exhaustion model demonstrated that chromatin accessibility at the BTG1 locus progressively increases during the establishment of exhaustion in tumors. This finding implicates BTG1 in reinforcing stress-adaptive or ‘quiescent-like’ transcriptional state in exhausted T cells, thereby suggesting that its function extends beyond the maintenance of naive T cell quiescence^89^.

## Perspective

Cellular quiescence, once perceived as a default state, is now robustly recognized as being intricately regulated by a myriad of molecular mechanisms. Likewise, in the context of T lymphocytes, considerable studies have identified several regulatory pathways associated with T cell quiescence, encompassing intrinsic, extrinsic and metabolic aspects. Interestingly, while the quiescence of T cells shares some features commonly observed in other types of quiescent cell, regulatory factors involved in the maintenance of T cell quiescence are selectively expressed in these cells and specialized to modulate T cell functions. Moreover, T cell activation, followed by stimulus upon TCR, leads to the decreased expression of these factors or inhibition of their activity, suggesting that these regulatory systems are evolutionarily programmed to optimize T cell quiescence. Intrinsic factors play a fundamental role in the maintenance of T cell quiescence by coordinating other regulatory signals from extrinsic and metabolic pathways, a multifaceted functionality handled in this Review Article^82^. In addition, naive T cells are characterized by a unique and distinct chromatin landscape, including bivalent modifications and super-enhancers to remain their identity and actively maintain quiescence while preparing for activation, distinguishing them from other states in T cell population^76,90^. Taken together, these regulatory processes ultimately contribute to maintaining the proper threshold for T cell activation and homeostasis in naive and memory pools. However, although growing information sheds light on new insights into T cell quiescence, details linking such scattered pathways depending on surrounding microenvironments, and how these factors are involved in different contexts of T cells still need to be further elucidated.

Recent studies have elucidated the roles of these quiescent factors in the context of T cell exhaustion or tumor control. For example, BTG1, which regulates cell cycle and proliferative state of T cells, appears to be re-expressed to impose quiescent characteristics on exhausted CD8^+^ T cells^89^. BACH2 limits the potential of differentiation by inhibiting access to the activation signatures, the expression of which supports the maintenance of stem-like CD8^+^ T cell population^77^. FOXP1 and KLF2 were recently identified as hub transcription factors within regulatory network that governs differentiation of CAR-T cells, where KLF2 promotes differentiation program into the effector program, suppressing exhaustion program, while FOXP1 promotes stemness and limits the transition of stem-like T cells to the effector subset^91^. Overexpression of another quiescent factor, FOXO1, in CAR-T cells enhances stem-like features, characterized by increased mitochondrial activity and improved memory fitness, ultimately leading to the effective tumor control^92,93^. These findings demonstrate that these factors can fulfill multifaceted roles across various stages of T cell differentiation, which extends beyond simply controlling naive T cell quiescence, a point previously emphasized primarily in the context of autoimmunity (Table 1).

**Table 1:** Summary of physiological phenotype and mechanism of quiescent factors related to the transcriptional regulation in naive T cells.

Target factors	Mechanism of action	Related phenotype		Disease context
KLF2	Regulation of genes related to the T cell migration (directly *Sell*, *S1pr1*) in naive T cell^47,50^ Regulation of inflammatory response with cytokine production and chemokine receptor (CXCR3) via cell autonomous and nonautonomous manner^45,47,52^	Decreased expression of T cell trafficking molecules and abnormal numbers and distribution of T cells in SLOs and peripheral tissues in cKO model^47,52^	Lymphopenia^46,47^ Increased risk in systemic lupus erythematosus by KLF2 enhancer variant^94^	
FOXO1	Enhanced survival of T cells by upregulating IL-7Rα and BCL-2 (refs. ^36,59^) Regulation of KLF2 and CCR7, which influence T cell trafficking^56^ Inhibition of transcription of AP-1 transcription factor family (*Junb, Fos, Fosb*) and upregulation of *Bach2*(ref. ^57^)	In cKO, increased CD44^hi^ effector^36^ and CD69^+^ population^59^ In cKO, increased cytokine and effector molecule expression (GZMB and IFN-γ)^57^ In cKO, increased apoptosis and decreased proliferation^57^	Autoimmune phenotypes in cKO model (production of autoantibodies and multiorgan infiltration)^36^ Colitis in bone marrow chimera model^36,59^ cKO CD8 T cell exhibit senescent phenotype^57^	
FOXP1	Downregulation of IL-7R expression, balancing its homeostasis with FOXO1 (ref. ^64^) Upregulation of Pik3ip1, which can inhibit PI3K–Akt–mTOR pathway^65^ Negative regulation of cell cycle through downregulation of E2F transcription factors (*E2f1*, *E2f3*and *Cdk1*)^65^	Reduced number of peripheral CD4^+^ and CD8^+^ T cells in mixed bone marrow chimera model^63^ In cKO, increased apoptosis in peripheral T cells, but not in the thymus^63^ Enhanced MER/ERK signaling, leading to the increased T cell activation in cKO^64^ Enhanced proliferation and progression into cell cycle in cKO^64,65^	Inverse correlation of FOXP1 expression with proliferation marker Ki-67 an increased survival with its high expression in peripheral T cell lymphoma^95^ Severe autoimmune phenotype in colitis and EAE models^96^	
BACH2	Negative regulation of super-enhancer signature genes, critical for T cell differentiation^76^ Inhibition of the action of AP-1 transcription factor family by occupying the same binding sites and limiting its accessibility^73^	Increased terminal differentiation and apoptosis of CD8^+^ T cells in cKO^73^ Increased helper T cell differentiation and cytokine production in cKO T cells^69^ (*This study used whole *Bach2* KO mice) Increased stem-like features in CD8^+^ T cells by its overexpression and decreased in cKO mice in exhaustion model^77^	Genetic mutations in various autoimmune diseases^70^ including asthma^71^, Crohn's disease^68^ and multiple sclerosis^72^	
BTG1 and BTG2	Control of mRNA stability and translation and downregulation of global mRNA abundance by deadenylation, thereby increasing the threshold for T cell activation^82^	Increased production of IL-2 and activation markers (CD44, CD69 and IL2RA) and enhanced proliferation in cKO^82^ Increased CD44^hi^CD62^lo^ effector memory population and decreased naive T cell population in cKO^82^	Missense mutation linked to the germinal center-derived B cell lymphoma^88^ Lymphomagenesis accelerated cKO^97^	

cKO, conditional knockout; IL-2, interleukin 2.

Maintaining a balance between quiescent versus activating states through the presence of cognate antigens and costimulatory signals is a crucial step for T cell-mediated immunity. Disruption of this balance leads to a loss of tolerance and aggressive immune responses by T cells, resulting in lymphoma, leukemia and various autoimmune disorders. Moreover, it has been suggested that molecules involved in maintaining T cell quiescence contribute to immune regulation in the tumor context. Clinical and immunological usefulness of T lymphocytes in infection clearance, vaccination, anticancer therapy and treatment of autoimmune diseases has facilitated great advances in T cell biology focused on effector function. However, more knowledge should be gained regarding quiescence in T cells. Dissecting the mechanisms underlying the maintenance of minimalism in quiescent T cells may provide fundamental clues for future applications in T cell-based therapies.
